# Effects of Diet and Strain on Mouse Serum and Tissue Retinoid Concentrations

**DOI:** 10.1371/journal.pone.0099435

**Published:** 2014-06-09

**Authors:** Kristin M. Obrochta, Maureen A. Kane, Joseph L. Napoli

**Affiliations:** Department of Nutritional Sciences and Toxicology, University of California, Berkeley, California, United States of America; Clermont Université, France

## Abstract

The relationship between dietary vitamin A and all-*trans*-retinoic acid levels in serum and tissues had not been quantified. We determined the impact of dietary vitamin A on retinoid levels in serum, liver, kidney, testis, and epididymal white adipose of five mouse strains: AKR/J; BALB/cByJ; C3H/HeJ; C57BL/6J; 129S1/SvImJ. Retinoids were quantified in mice fed copious vitamin A (lab chow, ≥20 IU/g) followed by one month feeding a vitamin A-sufficient diet (4 IU/g), or after three generations of feeding a vitamin A-sufficient diet. Retinol and retinyl esters were measured by high-performance liquid chromatography with ultraviolet absorbance detection. All-*trans*-retinoic acid was quantified by liquid chromatography tandem mass spectrometry. The amounts of dietary vitamin A had long-term strain-specific effects on tissue retinyl ester, retinol and all-*trans*-retinoic acid concentrations. Three generations of feeding a vitamin A-sufficient diet decreased all-*trans*-retinoic acid in most tissues of most strains, in some cases more than 60%, compared to a diet with copious vitamin A. With both diets, all-*trans*-retinoic acid concentrations maintained an order of liver ≈ testis > kidney > white adipose tissue ≈ serum. Neither retinol nor all-*trans*-retinoic acid in serum reflected all-*trans*-retinoic acid concentrations in tissues. Strain and tissue-specific differences in retinol and all-*trans*-retinoic acid altered by different amounts of dietary vitamin A could have profound effects on retinoid action. This would be the case especially with the increased all-*trans*-retinoic acid values associated with the amounts of vitamin A and its precursors (carotenoids) in chow diets.

## Introduction

Vitamin A (retinol) is essential for diverse physiological processes in post-natal vertebrates, including modulating energy metabolism, supporting neurogenesis and nervous system function, and sustaining the immune response [1]–[3]. As an autacoid derived from retinol, all-*trans*-retinoic acid (atRA) regulates transcription and translation by activating nuclear hormone receptors [4]–[6]. Retinol that exceeds demands for atRA production and for vision is stored as retinyl esters (RE), primarily in the liver, but also in many other tissues [7]. atRA auto-regulates its concentrations through substrate depletion *via* enhanced RE biosynthesis by inducing lecithin:retinol acyltransferase, and also through inducing its catabolism by inducing cytochrome P-450 isozyme expression [8], [9]. Excess or deficiency of atRA has adverse effects, such as abnormal fetal development and disruptions in regulation of energy balance [10], [11].

Most retinoid quantitation has been done on a few mouse strains, often fed standard laboratory chow. Standard rodent chow consists of natural sources, which vary in precise compositions, and contains copious amounts of nutrients, including vitamins [12]. In contrast, semi-purified diets provide a consistent nutrient composition in amounts that meet the National Research Council recommendations for rodent nutrient intake, while avoiding delivery of copious amounts of vitamins that can confound studies of nutrient functions. The American Institute of Nutrition (AIN) has determined that the minimum amount of vitamin A necessary for rodents is 2.9 IU/g diet, and recommends an intake of 4 IU/g diet to provide a margin of safety [13], [14]. Chow contains from 12 to >20 IU vitamin A/g diet, and likely provides even more depending on the carotenoid content of the natural products used to formulate the diets [15]. The upper limit of vitamin A for human consumption has been recommended as no more than 3–4 times the recommended dietary allowance (RDA) [16]. Vitamin A in rodent chow diet is ∼5 times the recommended intake for rodents, and therefore is exceptionally copious.

The present study focused on the impact of dietary vitamin A intake on retinoid status in five strains of mice. Mice bred from dams fed standard rodent chow were weaned onto a vitamin A-sufficient diet (VAS), the AIN93G semi-purified diet with 4 IU vitamin A/g [13]. After five weeks of VAS feeding, tissues of the first generation were collected and analyzed for RE, retinol and atRA concentrations. This first generation was used to breed a second generation, and the second was used to breed a third generation, while continuously being fed a VAS. Tissues and serum of the third generation were analyzed at a similar age as the first generation. The experimental design allowed for evaluation of the impacts of diet and strain on endogenous tissue retinoids.

## Materials and Methods

### Mice and diets

Mice were purchased from Jackson Laboratories at weaning (first generation). Mouse strains, abbreviations used, and catalog numbers are: 129S1/SvImJ (129) (002448); C3H/HeJ (C3H) (000659); BALB/cByJ (BALB) (001026); AKR/J (AKR) (000648); C57BL/6J (C57) (000664). First generation mice had been bred from and nursed by dams fed a chow diet (Lab Diet JL 6%, catalog 5K0Q), which contained copious amounts of vitamin A, as retinol, RE, and β-carotene (≥20 IU total vitamin A/g diet). Upon arrival, mice were fed an AIN93G semi-purified diet (Dyets, catalog 110700) with sufficient vitamin A (4 IU/g diet) in the form of retinyl palmitate. The VAS was maintained throughout the study for all mice (dams and pups), including during breeding, nursing, and after weaning. Mice were housed up to five per cage with littermates, and fed ad libitum for the duration of the study. Mice were euthanized in the morning, at 9–10 weeks old. This study was done in strict accordance with the recommendations in the Guide for the care and use of Laboratory Animals of the National Institutes of Health. The protocol was approved by the Animal Care and Use Committee of UC-Berkeley.

### Quantification of retinoids

Retinoid concentrations were quantified in 6 to 10 male mice per group. Tissues were collected under yellow light, weighed, and frozen immediately in liquid nitrogen. Tissues were thawed on ice and hand homogenized in 0.9% saline. Retinoids were recovered by a two-step acid and base extraction [17]. All materials in contact with samples were glass or stainless steel. Internal standards were used to calculate extraction efficiency of retinoids. RE and retinol were extracted and then quantified by HPLC-UV as described, with retinyl acetate as internal standard [18]. atRA was extracted and quantified by LC/MS/MS as described, with 4,4- dimethyl atRA as internal standard [19], [20].

### Statistics

Data are presented as mean ± standard error (SE) and were analyzed using two-tailed, unpaired student's *t* tests or linear regression analysis.

## Results

### Serum and tissue RE concentrations

**Figure 1:**
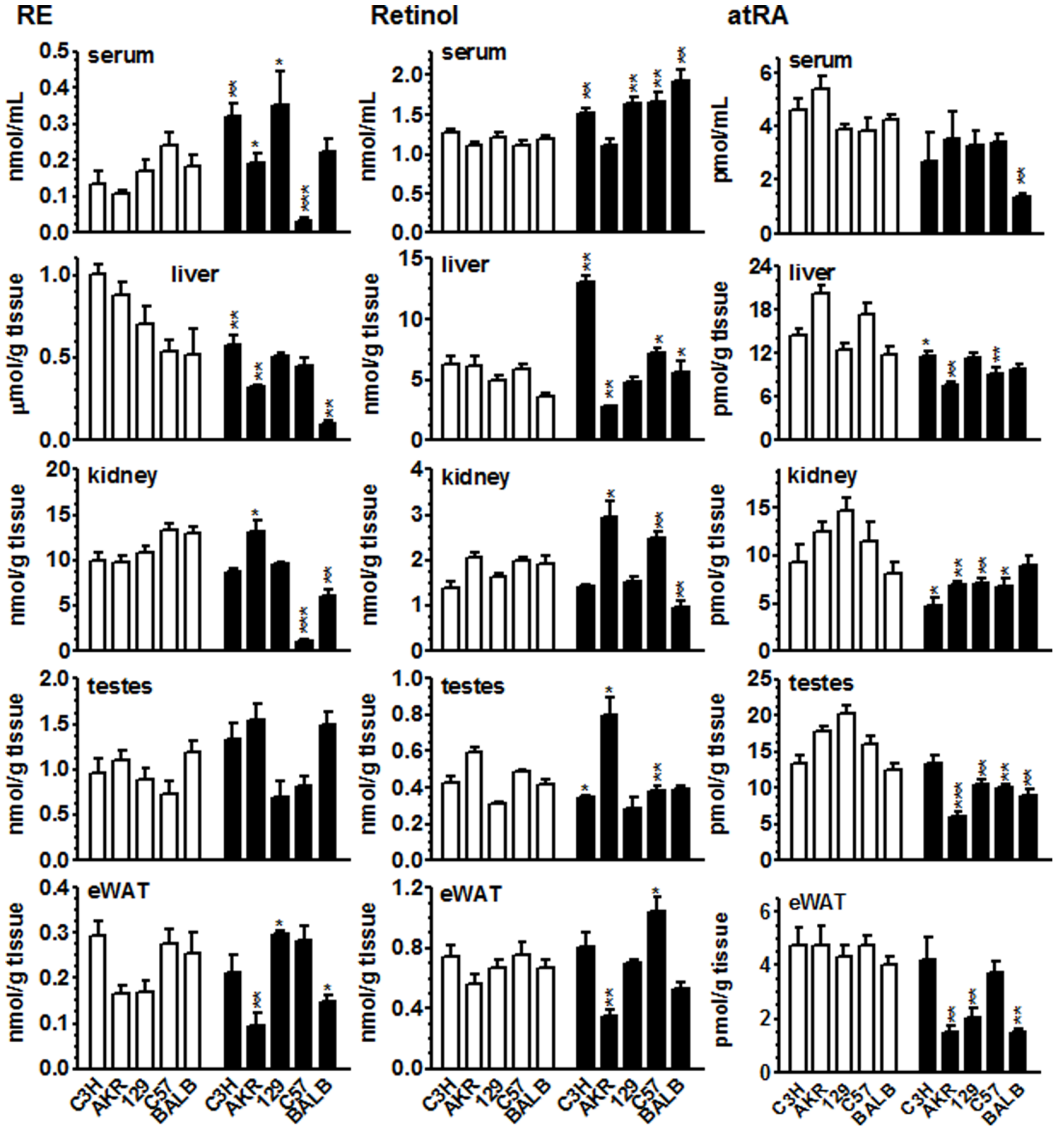
Retinoids in serum and tissues of five mouse strains. Retinyl esters (RE), retinol and all-*trans*-retinoic acid (atRA) in serum and tissues. Mice were fed a VAS after weaning from dams fed a chow diet (generation 1). Generation 1 mice were maintained and bred on the VAS, continuing into the third generation. Retinoids were quantified in male mice after week 9 of the first generation (open bars), or week 10 of the third generation (filled bars). Tissues and serum of 3 to 10 mice were assayed for each strain and generation: eWAT, epididymal white adipose tissue. Data are means ± SE: *P<0.05; **P<0.005, ***P<0.001 relative to first generation values.

Serum RE concentrations occurred in a limited range from 0.1 to 0.24 nmol/ml in the first generation mice switched to a VAS (Figure 1). By the third generation of VAS feeding, serum RE in the C3H, AKR and 129 strains increased ∼2-fold, decreased 86% in C57 mice and did not change significantly for BALB mice, with a range from 0.2 to 0.35 nmol/ml for the five strains. Liver RE concentrations were greater in first-generation than in third generation mice for all but C57 strain, and spanned a wide range (∼360 to 1000 nmol/g tissue). RE in the third generation of C3H, AKR and BALB mice decreased 42, 64 and 73%, respectively, relative to the first generation. In the 129 strain, the 27% decrease was not statistically significant (*P* = 0.3). Liver RE of C57 were unaffected by long-term VAS. The range of liver RE in the third generation varied from ∼100 to 580 nmol/g tissue). Kidney RE concentrations were similar regardless of strain for first-generation mice, ranging from ∼10 to 13 nmol/g tissue, and remained the same for the VAS-fed C3H and 129 strains into the third generation. RE in kidney of the third generation mice increased 34% in AKR, and decreased 92 and 54% in C57 and BALB mice, respectively. The range for kidney RE in the third generation was broad (1 to 13.3 nmol/g tissue). Testes RE concentrations occurred in a tight range regardless of strain in first-generation mice (0.7 to 1.2 pmol/g tissue), and did not change significantly in mice fed a VAS into the third generation (0.8 to 1.5 pmol/g tissue). RE concentrations in white adipose were the lowest of the tissues assayed, ranging from ∼0.17–0.3 nmol/g in first-generation mice. Long-term VAS did not significantly change white adipose RE in C3H and C57 strains, but resulted in a 1.8-fold increase in 129, and >60 and 40% decreases in AKR and BALB strains, respectfully. The range of RE in white adipose, 0.15 to 0.3 nmol/g tissue, was similar for third generation as first generation mice fed VAS.

### Serum and tissue retinol concentrations

Serum retinol concentrations were nearly identical regardless of mouse strain in first-generation mice fed a VAS, ranging from 1.1 to 1.3 nmol/ml (Figure 1). Serum retinol increased 18, 35, 50, and 60%, respectively, for long-term VAS-fed C3H, 129, C57 and BALB strains, but did not change in the AKR strain. A VAS broadened the range to span from 1.1 to 1.7 nmol retinol/ml. Liver retinol concentrations spanned a limited range of 3.6 to 6.9 nmol/g tissue in first-generation mice. Long-term feeding a VAS diet increased liver retinol 25 to 100% in C3H, C57 and BALB strains, decreased retinol 55% in AKR, and did not change retinol in 129 mice. This resulted in a much broader range of liver retinol values, 2.8 to 13 nmol/g tissue, compared to the first generation. Kidney retinol concentrations spanned a limited range of 1.4 to 2.1 nmol/g tissue in first-generation mice, and varied widely with strain in response to long-term VAS-feeding, remaining the same in the C3H and 129 strains, increasing 38 and 26% in AKR and C57 strains, respectively, and decreasing 50% in BALB. This resulted in an expanded range of ∼1 to 2.5 nmol/g tissue. Testis retinol concentrations ranged from 0.3 to 0.6 nmol/g tissue in first-generation mice, and also reacted in a strain-dependent manner to long-term VAS-feeding, resulting in no change for 129 and BALB mice, a ∼20% decrease in C3H and C57 mice, a 33% increase in AKR. The range was only modestly affected, however (0.3 to 0.8 nmol/g tissue), in third generation mice. White adipose retinol concentrations were very close regardless of mouse strain in first-generation mice, ranging tightly from ∼0.6 to 0.8 nmol/g tissue. Long-term VAS did not significantly affect white adipose retinol concentrations in C3H, 129, or BALB strains, but produced a 39% decrease in AKR, and a 38% increase in C57. The range of white adipose retinol broadened to 0.4 to 1 nmol/g tissue.

### Serum and tissue atRA concentrations

Serum atRA concentrations were similar in the five strains in the first generation, ranging from 3.8 to 5.4 pmol/ml (Figure 1). Long-term feeding a VAS did not alter serum atRA significantly, except for BALB mice, which responded with a 68% decrease. Liver atRA concentrations decreased with long-term VAS feeding 21, 62, and 53% in C3H, AKR, and C57 strains, respectively, but did not change in 129 and BALB mice. The range of liver atRA narrowed from ∼9 to 24 pmol/g tissue in first generation to ∼8 to 13 pmol/g tissue with long-term VAS. Kidney atRA concentrations in first generation mice ranged from 7 to 13 pmol/g tissue. Long-term VAS caused kidney atRA to decrease from 37 to 52% for all but the BALB strain, which remained unaffected. This resulted in a reduced range of ∼5 to 9 pmol/g tissue. Testis atRA concentrations ranged from 13–20 pmol/g tissue in first generation strains, *i.e.* similar to the values in liver. Long-term feeding VAS decreased testis atRA 30 to 66% for all strains except C3H, which was unaffected. The range of testis atRA reduced to ∼6 to 13 pmol/g tissue—again similar to concentrations measured in liver. White adipose atRA concentrations were very similar among strains in the first generation assayed, ranging tightly from 4 to 4.8 pmol/g tissue, with an average value of 4.5 pmol/g tissue. Long-term feeding VAS did not change white adipose atRA in the C3H and C57 strains, but decreased it 69, 53 and 62% in the AKR, 129 and BALB strains, respectfully. The strain-specific response to VAS increased the range of white adipose atRA to ∼1.5 to 4.2 pmol/g tissue.

atRA generally decreased in tissues of mice fed the VAS diet for three generations, with 14 of 20 tissues in the five strains showing significant decreases. atRA did not change significantly in the remaining 6 tissues, and in no case did it increase with VAS. Although the effects of reducing the amount of dietary vitamin A on tissue atRA were both strain and tissue dependent, the relative orders of atRA tissue concentrations remained the same in the first and third generations. Liver and testis had the highest concentrations, kidney had intermediate concentrations, and white adipose had the lowest concentrations.

**Figure 2:**
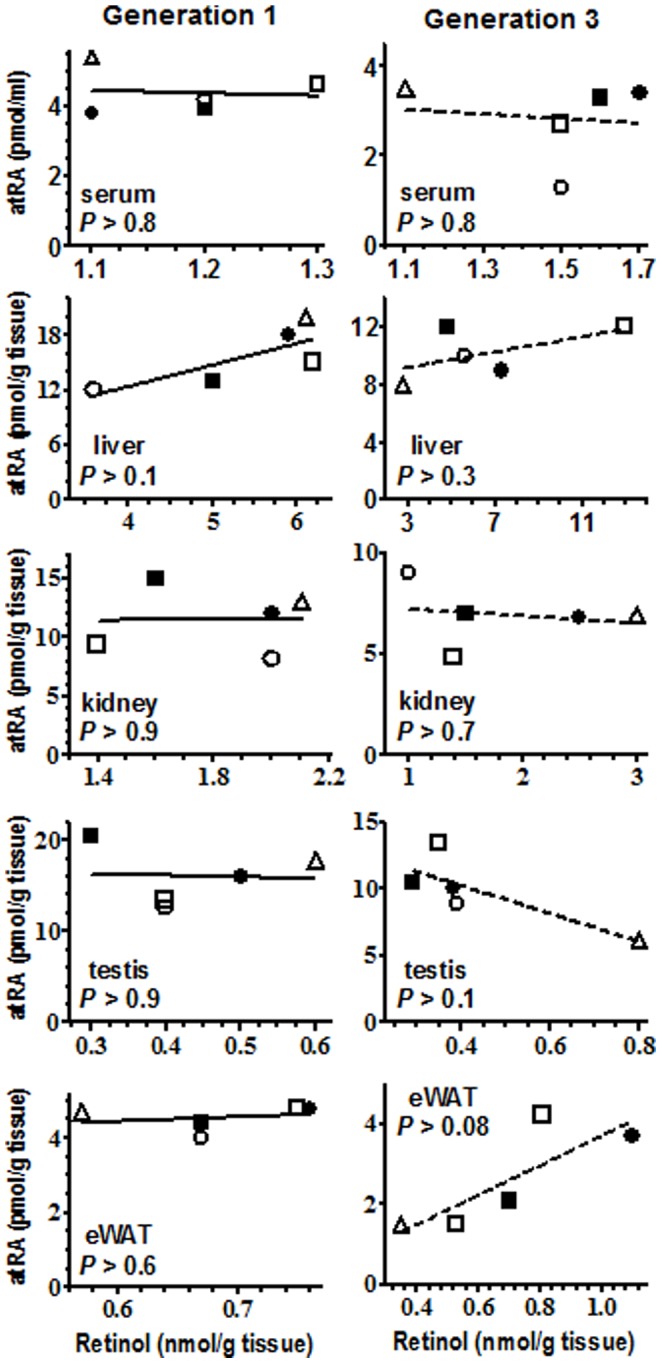
Relationships between retinol and atRA. Retinol (x-axis) and atRA (y-axis) values for serum and tissues from first generation (left column) and third generation (right column) fed a VAS (eWAT, epididymal white adipose tissue): □, C3H; Δ, AKR; ▪, 129; •, C57; ◯, BALB. Linear regression analysis for each tissue and generation verified that none of the slopes differed significantly from zero.

Serum atRA values clustered in both the first and third generations (except for BALB in the third generation) and therefore were dependent on diet rather than strain. This clustering of atRA values in serum contrasts with tissue-specific variations, and indicates that serum atRA does not reflect or predict tissue atRA concentrations. Neither did serum retinol reflect tissue atRA. Further, no linear correlations were observed between retinol and atRA concentrations in serum or tissues from either generation (Figure 2). Linear regression analysis of each data set revealed none with a slope significantly different from zero.

## Discussion

Previous reports with mice fed copious vitamin A have shown that liver RE correlate directly with dietary vitamin A levels when mice are challenged with even greater dietary vitamin A, or fed a vitamin A-deficient diet [21], [22]. The impact, however, of transitioning from a chow diet to a VAS had not been studied, nor had strain differences been addressed. The present study addressed these issues, and revealed that the transition of copious to sufficient dietary vitamin A affects retinoid homeostasis with strain and tissue-specificity. The decreases in atRA with a decrease in dietary vitamin A across the five strains show that retinol availability affects atRA concentrations, even with modest changes in dietary vitamin A, but the lack of correlation between retinol and atRA concentrations indicates that strain-specificity determines the exact relationships between the two. Although atRA decreased in general with a decrease in dietary retinol, sometimes greater than 60%, the magnitudes of the decreases did not adhere to quantitatively predictable patterns. This partial dependence of atRA on dietary vitamin A, and its strain and tissue-dependent variation, would have physiological consequences because relatively modest 2-fold increases in atRA concentrations can have toxic effects [10]. Even in the absence of toxicity, increased atRA *via* increased dietary retinol may have profound consequences. Phenotypes resulting from ablation of genes that modulate retinoid homeostasis can be rescued by a chow diet (copious vitamin A). For example, when fed chow diet *Rbp4*-null (*Rbp4* encodes the serum retinol binding-protein) mice reproduce and grow normally, experiencing only modestly impaired vision, but when fed a VAS diet fail to thrive and reproduce [23]. Similarly, *Rdh1*-null mice fed a chow diet do not display a phenotype, but when fed a VAS become ≥30% heavier than wild-type littermates, even when fed a low-fat diet [24]. *Rbp2*-null (*Rbp2* encodes cellular retinol binding-protein type 2) mice nursed by dams fed a chow diet show no phenotype, but when nursed by dams fed a diet limited in vitamin A have high neonatal mortality [25]. These observations are consistent with evolution of retinoid homeostasis associated-proteins enabling animals to efficiently use and thrive on limited dietary vitamin A. Copious dietary vitamin A may allow synthesis of atRA by non-physiological paths, independent of retinoid-chaperoning proteins, and thereby would obscure the functions of proteins that evolved to increase the efficiency of vitamin A use. Even with phenotypes that manifest in mice fed a diet with copious vitamin A, the higher vitamin A levels of chow diets might ameliorate the severity of the phenotype or reduce its complexity. Because atRA has multiple actions in diverse tissues, and laboratory mice are rarely challenged to the extent of mice living in the wild, the ameliorating impact of a chow diet on a knock-out would not necessarily be obvious. The combination of a protected environment and a chow diet can reasonably be expected to prevent manifestations of phenotypes, and therefore could render a gene ablation non-informative.

Surprisingly, not all strains responded to decreased dietary retinol with a decrease in liver RE. Two of the most popular strains used to ablate genes, 129 and C57, did not experience significant decreases in liver RE over three generations of feeding a VAS. This phenomenon of conserving RE, even during reduced dietary intake, likely underlies the common observation that mice are difficult to render vitamin A deficient, if bred from dams fed a diet copious in vitamin A. Other results also were not anticipated, such as the C57 strain having a very large decrease in kidney RE during long-term VAS feeding, while liver RE resisted change. The strain-specific effect of diet on white adipose RE and the lack of correlation with liver RE also were not anticipated. Increases in serum RE and retinol with decreases in dietary vitamin A in four of five strains also were not anticipated, and perhaps reflect enhanced mobilization from liver to support extra-hepatic tissue atRA biosynthesis. The inconsistent changes in tissue retinol with decreases in dietary retinol were another unexpected outcome, and indicate that tissue retinol concentrations were more strain than diet dependent in mice fed a VAS.

Serum atRA exchanges with tissue atRA, except in testis [26]. The blood-testis barrier prevents physiological amounts of circulating atRA from entering Sertoli and germ cells. Instead, retinol permeates the blood-testis barrier and supports atRA biosynthesis *in situ*. Therefore, testis atRA would most likely reveal the true nature of the relationship between tissue retinol and atRA, compared to other tissues. The amount of dietary retinol affected the steady-state concentrations of atRA in testis in a strain-specific final concentration, just as with other tissues, which reinforces strain dominance of the retinol-atRA relationship (except for the AKR strain).

Strain dependent retinoid concentrations suggest differential activity and/or expression of enzymes that catalyze retinoid homeostasis. Biosynthesis and catabolism of atRA are regulated to meet biological demand and remain within a safe range. Retinol from diet, or mobilized from RE by retinyl ester hydrolases is substrate for atRA biosynthesis [27]. Two sequential reactions from retinol catalyze synthesis of atRA. Retinol dehydrogenases (Rdh) catalyze the first and rate limiting reaction that converts retinol into retinal. Retinal dehydrogenases (Raldh) catalyze the conversion of retinal into atRA [9]. Various cytochromes P-450 (Cyp) catabolize atRA [8]. Multiple isozymes of Rdh, Raldh and Cyp contribute to atRA biosynthesis and catabolism. These data also indicate that the concentrations of atRA are tissue autonomous, because the relative amount in each tissue differs with strain. For example, the relative order of atRA in third generation VAS liver was C3H ≈ 129 > BALB > C57 > AKR, but in kidney was BALB > AKR ≈ 129 ≈ C57 > C3H. This observation suggests that a comparison of retinoid metabolizing enzyme expression in tissues of various strains could provide insight into the enzymes that predominate in maintaining atRA homeostasis.

Understanding the impact of dietary vitamin A on retinol and atRA levels would be important to understanding retinoid homeostasis and to the study of animals with disruptions in retinoid homeostasis-regulating genes. This work showed that: 1) copious vitamin A, such as used in chow diets, increases tissue atRA markedly; 2) decreasing dietary retinol decreases serum and tissue atRA with strain-specific degrees; 3) neither serum retinol nor atRA reflect tissue atRA concentrations; 4) RE concentrations react to a decrease in dietary vitamin A with strain and tissue-specific effects; 5) replacing a copious vitamin A diet (chow) with a VAS for one month (generation 1) did not reduce serum and tissue atRA levels to those achieved with long-term VAS feeding (generation 3). These data quantify the impact of dietary vitamin A on endogenous retinoid concentrations, and highlight both strain and tissue-specific differences that could have profound effects on retinoid action. Notably, elevated atRA in tissues of mice fed copious vitamin A could rescue or mask phenotypes generated by ablating retinoid homeostasis-regulating genes.
